# Genome-Wide Characterization of the Wheat Pum Gene Family and Heterologous Functional Analysis of *TaPum-III-5b* in *Arabidopsis* Under Salt Stress

**DOI:** 10.3390/plants15192912

**Published:** 2026-09-23

**Authors:** Wenjie Zheng, Zhengyong Cui, Peng Li, Miao Wang, Chen Huang, Wei Li, Xin Sun, Ying Liu, Mingzhu Sun, Baiqiang Yan

**Affiliations:** 1 Crop Research Institute, Shandong Academy of Agricultural Sciences/National Engineering Research Center of Wheat and Maize/State Key Laboratory of Wheat Improvement/Key Laboratory of Wheat Biology and Genetic Improvement in North Yellow & Huai River Valley/Shandong Provincial Technology Innovation Center for Wheat, Jinan 250100, China; 2 Shandong Luyan Seed Co., Ltd., Jinan 250100, China

**Keywords:** RNA-binding protein, pumilio gene family, *TaPum-III-5b*, *TaDREB2B*, salt stress

## Abstract

Pumilio (Pum)-family proteins are evolutionarily conserved RNA-binding proteins that regulate post-transcriptional processes; however, their specific functions in staple crops, for example, wheat (*Triticum aestivum*), remain poorly understood. We identified 56 *TaPum* genes and classified them into three phylogenetic subgroups (I–III). The larger *TaPum* family size in allohexaploid wheat was associated with retention of A-, B-, and D-subgenome homoeologs, segmental duplication, and tandem duplication. After separating homoeologous pairs, 17 segmental duplication pairs and five tandem duplication pairs were identified, and ratios of nonsynonymous to synonymous substitutions (Ka/Ks) below 1 were consistent with purifying selection. Subgroup-specific motifs and the uneven distribution of cis-acting elements, particularly abscisic acid (ABA)- and methyl jasmonate (MeJA)-responsive elements, suggested potential divergence in promoter regulatory architecture. Transcriptomic profiling and independent quantitative reverse transcription-polymerase chain reaction analysis showed more consistent response of *TaPum* genes to salt stress than to cold or drought stress. Salt stress strongly promoted *TaPum-III-5b* expression, and its encoded protein exhibited nuclear and cytoplasmic localization. Heterologous overexpression of *TaPum-III-5b* in *Arabidopsis thaliana* increased salt tolerance, as indicated by enhanced survival; augmented peroxidase, superoxide dismutase, and catalase activities; and diminished accumulation of reactive oxygen species and malondialdehyde. Electrophoretic mobility shift assay (EMSA) demonstrated sequence-specific binding of *TaDREB2B* to a dehydration-responsive element (DRE)-containing sequence in the *TaPum-III-5b* promoter, whereas dual-luciferase (Dual-LUC) analysis showed activation of the wild-type promoter. Together, these results support a proposed regulatory framework in which *TaPum-III-5b* may contribute to salt-stress responses through as-yet-unidentified downstream RNA targets. This study characterizes the Pum gene family in wheat and identifies the *TaDREB2B-TaPum-III-5b* module as a candidate regulatory pathway associated with salt-stress responses. Its potential application in wheat improvement requires further validation in wheat.

## 1. Introduction

RNA-binding proteins (RBPs) mediate post-transcriptional gene regulation. They regulate several processes, including RNA splicing, stability, modification, transport, translation, and localization. RBPs coordinate RNA metabolism by recognizing specific RNA structures or sequences and interacting with protein partners [1,2]. Pumilio (Pum) is an evolutionarily conserved RBP and a key post-transcriptional regulator widely distributed among eukaryotes. The name “Pumilio” is derived from the Latin for “little one” or “dwarf” [3].

Pum family proteins, also known as Pumilio and fem-3 messenger RNA (mRNA)-binding factor (PUF) proteins, contain tandem repeats that form a conserved Pumilio homology domain (Pum-HD). The Pum-HD recognizes a conserved 8- to 10-nucleotide core sequence containing the UGUA tetranucleotide motif, typically located in the 3′-untranslated region (UTR) of target mRNAs, thereby regulating mRNA stability and translation [4,5]. Pum-mediated repression occurs through several mechanisms, including the recruitment of deadenylases, which promote translational repression [6], and the possible inhibition of ribosome translocation through interactions with translation initiation factors [7]. Some Pum isoforms also repress target transcripts through coordinated pathways that involve microRNAs and potentially other Pum proteins [8]. Pum isoforms are present in various organisms. *Caenorhabditis elegans* possesses 11 Pum homologues [9], while *Trypanosoma cruzi* contains 10 Pum genes classified into three groups according to their predicted binding preferences [10]. PfPuf1 and PfPuf2 isoforms in *Plasmodium falciparum* have been reported. Additionally, two Pum genes have been detected in *Oncorhynchus mykiss* and chicken genomes each [11,12], and two putative Pum genes, namely *TgPuf1* and *TgPuf2*, have been identified in *Toxoplasma gondii* [13]. Although Pum-family proteins are relatively well studied in animals and fungi [14,15], their functions in plants remain poorly understood.

Research on the plant Pum family remains at an early stage. The genomes of *Arabidopsis thaliana*, *Arabidopsis lyrata*, apple (*Malus domestica*), maize (*Zea mays*), soybean (*Glycine max*), japonica rice (*Oryza sativa* ssp. *japonica*), and indica rice (*Oryza sativa* ssp. *indica*) contain 26, 31, 28, 19, 26, 20, and 22 Pum genes, respectively [16,17,18,19]. Compared to the numbers reported in animals, this relative expansion suggests that plant Pum proteins participate extensively in post-transcriptional regulation and may help plants respond to environmental stresses. However, studies on plant Pum genes remain scarce and have focused primarily on *Arabidopsis*. AtPUM1–6 proteins function as post-transcriptional regulatory hubs with roles in balancing stress tolerance and growth as well as embryonic development [20]. In particular, AtPUM5 binds to the 3ʹ-UTRs of abiotic stress-responsive genes containing Pum RNA-binding motifs to negatively regulate resistance to stress due to drought and salinity; it also responds to cucumber mosaic virus infection [21,22]. *AtPUM9* is specifically expressed during seed maturation and imbibition and promotes germination by negatively regulating abscisic acid (ABA) signaling genes. Its overexpression causes abnormal leaf development, delayed flowering, and enhanced thermotolerance, reflecting its diverse roles in development and stress adaptation [23]. AtPUM23 and AtPUM24 show nucleolus localization and have critical functions in ribosomal RNA (rRNA) processing and in early embryogenesis and tolerance to salt stress, respectively [24,25]. Nevertheless, the functions of most plant Pum proteins remain largely unexplored.

Salt stress, a severe form of abiotic stress, exerts cumulative adverse effects throughout the plant life cycle. It inhibits the growth of plants, reduces crop yield, threatens biodiversity, accelerates desertification, and creates socioeconomic problems [26,27]. Salt exposure rapidly activates the defense mechanisms of plants. Osmoprotectants, including soluble sugars and proline, accumulate to maintain cellular integrity; antioxidant enzymes, including catalase (CAT), peroxidase (POD) and superoxide dismutase (SOD), increase to limit reactive oxygen species (ROS) accumulation and subsequent oxidative damage [28,29]. Salt stress also activates transcription factors (TFs) that coordinate systemic defense responses [30,31]. Plants possess numerous TF families, such as the NAC (named after NO APICAL MERISTEM, *ARABIDOPSIS* TRANSCRIPTION ACTIVATION FACTOR 1/2, and CUP-SHAPED COTYLEDON 2), APETALA2/ethylene-responsive factor (AP2/ERF), WRKY, and MYB, which exhibit substantial structural and functional diversity and contribute to plant growth, development, and stress tolerance [32].

The production of wheat (*Triticum aestivum*), a major global staple crop, is highly vulnerable to environmental stress. Salt stress, in particular, can severely impair wheat growth and development and cause substantial yield losses [33]. Previous reports led us to hypothesize the involvement of wheat Pum genes in salt-stress response. However, there are no systematic studies on the wheat Pum gene family. We therefore investigated the wheat Pum gene family in this study. We found 56 *TaPum* genes and examined their chromosomal locations, *cis*-acting regulatory elements, conserved protein domains, phylogenetic relationships, physicochemical properties, and expression patterns during salt stress. We also conducted a preliminary functional characterization of *TaPum-III-5b*. This study aimed to clarify how *TaPum* genes influence the wheat plant’s response to salt stress; the findings provide a basis for characterizing Pum gene families in plants.

## 2. Results

### 2.1. The Wheat Pum Gene Family: Genome-Wide Identification and Characterization

To determine evolutionary relationships among Pum proteins across species, Pum protein sequences from wheat and previously reported sequences from *Arabidopsis* (26 proteins) and maize (19 proteins) were used to construct a phylogenetic tree. The Pum proteins were assigned to three subgroups (Figure 1). Among the wheat Pum proteins, 26 were assigned to subgroup I, 12 to subgroup II, and 18 to subgroup III. In contrast, most *Arabidopsis* Pum proteins belonged to subgroup II, with subgroups I, II, and III containing 6, 17, and 3 proteins, respectively. The contrasting subgroup distributions among species indicate lineage-specific diversification of the Pum family and suggest potential functional divergence among evolutionary lineages. Although the three major subgroups were recovered, 10 of the 98 internal nodes had bootstrap support below 50%; relationships involving these weakly supported branches and the associated subgroup-specific interpretations should therefore be treated cautiously.

The wheat Pum family members were designated *TaPum-I-1a* to *TaPum-III-9d* according to their chromosomal group and subgenome designation (Supplementary Table S1). Physicochemical analysis showed that the lengths and molecular weights of the predicted TaPum proteins ranged from 350 to 1052 amino acids and from 39.23 kDa (TaPum-III-6d) to 115.07 kDa (TaPum-I-11a), respectively. Their theoretical isoelectric points (pIs) ranged from 5.25 (TaPum-I-8a) to 9.5 (TaPum-III-3b). Their instability indices ranged from 32.52 (TaPum-II-3a) to 58.92 (TaPum-III-1b), and their aliphatic indices ranged from 67.93 (TaPum-I-8a) to 102.54 (TaPum-III-6d). According to grand average of hydropathicity values, all TaPum proteins were considered hydrophilic, although TaPum-III-6d exhibited only weak hydrophilicity (Supplementary Table S2).

Structural modelling of representative Pum proteins from each phylogenetic group in wheat, *Arabidopsis*, and maize revealed a compact, alpha-helical Pum-HD core across all nine proteins. The predicted Pum-HD folds were broadly similar among representatives, whereas the flanking regions varied in length and spatial arrangement, with extended coil-rich segments apparent in several models. Because these peripheral features were model predictions, their apparent structural differences were interpreted cautiously (Supplementary Figure S1; Supplementary Table S6).

### 2.2. Structural Features of TaPum Genes and Proteins

From the wheat genome, 56 *TaPum* family members were identified and phylogenetically classified into three unevenly represented subgroups, designated Groups I–III. Members within the same phylogenetic clade exhibited high sequence similarity, consistent with conservation of their core sequences (Figure 2A).

Based on Multiple EM for Motif Elicitation (MEME) analysis, 10 conserved motifs were detected among the TaPum proteins. Motifs 1 and 2 were present in 87.5% of the family members and were therefore considered core motifs. More than 72% of TaPum proteins contained 6 to 10 conserved motifs, whereas less than 17.9% contained fewer than 4 motifs. Motif composition and arrangement were similar among members of the same phylogenetic subgroup. Each subgroup also exhibited a distinct motif combination, indicating subgroup-specific motif organization (Figure 2B).

Domain annotation showed that all TaPum proteins contained the characteristic Pumilio domain. In addition, 41.1% contained auxiliary domains, including members of the nucleic acid-binding protein (NABP) and PHA03247 superfamilies and the Zf-XS domain, indicating domain diversity beyond the core RNA-binding activity (Figure 2C).

According to gene structure analysis, *TaPum* genes varied substantially in their length. The number of coding sequence (CDS) regions per gene was 5–18, and 8.9% of the genes lacked both 5′- and 3′-UTRs. The same phylogenetic clade members had similar exon–intron structures; in contrast, those belonging to different subclades showed clear structural variation (Figure 2D).

### 2.3. Collinearity and Chromosomal Distribution of TaPum Genes

To examine *TaPum* gene family expansion and evolutionary history, interspecific and intraspecific collinearity among the 56 identified genes was analyzed. Interspecific synteny analysis identified one orthologous gene pair between *Arabidopsis* and wheat and 31 pairs between maize and wheat (Figure 3A). Within the wheat genome, a total of 25 gene pairs were classified as A/B/D homoeologous pairs, 17 gene pairs as segmental duplication pairs, and five gene pairs as tandem duplication pairs (Figure 3B; Supplementary Table S3). The five tandem duplication pairs are TraesCS2B02G449300.1–TraesCS2B02G449400.1, TraesCS4A02G364800.1–TraesCS4A02G364900.1, TraesCS5B02G508600.1–TraesCS5B02G508700.1, TraesCS5D02G508500.1–TraesCS5D02G508600.1, and TraesCS5D02G508600.1–TraesCS5D02G508700.1. To determine the selective pressures influencing the duplicated genes, the nonsynonymous to synonymous substitution (Ka/Ks) ratio was calculated. All duplicated *TaPum* gene pairs showed Ka/Ks values below 1, indicating that their evolutionary patterns were consistent with purifying selection (Supplementary Table S3).

Chromosomal mapping indicated uneven distribution of the 56 *TaPum* genes across the 21 wheat chromosomes. The largest number of *TaPum* genes was noted in Chromosome 5D, with seven members, whereas chromosomes 1A, 1B, 1D, 7A, 7B, and 7D each contained only one. Most *TaPum* genes were positioned close to the chromosomes’ terminal regions (Figure 3B; Supplementary Figure S2).

### 2.4. Cis-Acting Regulatory Elements in TaPum Promoters

After retrieving DNA sequences (2 kb) upstream of the coding regions of the 56 wheat *TaPum* genes, *cis*-acting regulatory elements were screened in these sequences using the PlantCARE database. We identified 11 *cis*-acting element classes and assigned them to the following functional groups: hormone responsiveness, growth and developmental regulation, and stress responsiveness (Figure 4). These elements were unevenly distributed among genes and phylogenetic subgroups.

Light-responsive elements were most abundant (618), constituting more than 40% of all detected *cis*-acting elements. ABA- and methyl jasmonate (MeJA)-responsive elements were the predominant hormone-responsive elements, with 233 and 250 elements, respectively, providing predictive, rather than functional, evidence concerning *TaPum* genes in ABA- and MeJA-mediated stress response pathways. MeJA-responsive elements were particularly abundant in subgroup III. For example, *TaPum-III-1b* contained 16 MeJA-responsive elements, whereas most members of subgroups I and II contained 0 to 6, highlighting subgroup differences in predicted promoter composition rather than establishing functional specialization of subgroup III in jasmonate signaling.

Stress- and development-related elements were more broadly distributed, although several genes showed distinct enrichment patterns. For example, *TaPum-II-1a*, which contained the fewest *cis*-acting elements overall, harbored seven drought-responsive elements. Cold-responsive elements were particularly abundant in *TaPum-III-1b* (nine elements) and *TaPum-I-8a* (five elements), highlighting differences in their predicted promoter architectures (Supplementary Table S4). Overall, the diverse *cis*-acting element profiles of the *TaPum* promoters provide testable hypotheses regarding potential regulatory differences among *TaPum* genes that require experimental validation.

### 2.5. TaPum Gene Expression Pattern in Tissues and During Abiotic Stress

To examine potential functions of the wheat *TaPum* gene family, we evaluated transcriptome-wide expression profiles in diverse tissues and during several abiotic stress treatments; WheatOmics 1.0 (https://wheatomics.sdau.edu.cn/expression/; date of access: 12 January 2025) was used for this analysis. We found very low transcript abundance for most *TaPum* genes in roots, stems, and leaves across the developmental stages examined. In contrast, many *TaPum* homoeologs were strongly expressed in spikes and developing grains, suggesting potential roles in spike differentiation and grain development. A small number of genes, including *TaPum-II-3a* and *TaPum-II-5d*, showed tissue-specific expression in stems and leaves, consistent with possible roles in vegetative growth (Figure 5A).

Under cold stress, *TaPum* genes formed two major expression clusters. Genes in the upper cluster were markedly repressed by low temperature, whereas those in the lower cluster showed substantially higher expression at 4 °C than under control (CK) conditions (Figure 5B).

The temporal expression trends of *TaPum* genes were assessed during heat, drought, and combined drought–heat stress (Figure 5C). *TaPum-II-4a/4b* and *TaPum-I-10a* were specifically induced after 1 h of drought treatment and 6 h of heat treatment, whereas *TaPum-I-3a/d* and *TaPum-II-2a* were specifically induced after 6 h of drought treatment. Several genes, including *TaPum-III-8a/b/d*, were upregulated under heat stress.

In contrast, most *TaPum* genes were strongly induced by salt stress. Several members, including *TaPum-III-5a* and *TaPum-I-7d*, showed sustained induction after 6, 12, and 24 h of salt exposure, whereas a smaller subset, including *TaPum-III-1a* and *TaPum-II-2a*, reached high transcript levels only after 24 h. Overall, the *TaPum* family displayed a more consistent and coordinated transcriptional response to salt stress than to the other abiotic stresses examined (Figure 5D).

To independently examine whether representative stress-response trends were also observed in Luyan951, quantitative reverse transcription polymerase chain reaction (qRT-PCR) analyses were performed after cold and salt treatments. Nine stress-responsive *TaPum* genes representing subgroups I, II, and III were examined at 0, 6, 12, and 24 h (Figure 6).

Under cold stress, several examined *TaPum* genes showed time-dependent induction, whereas others exhibited weak or nonsignificant responses. *TaPum-I-2a*, *TaPum-I-2b*, *TaPum-I-10a*, and *TaPum-II-1d* showed sustained upregulation from 6 to 24 h. Among these genes, *TaPum-II-1d* exhibited the strongest cold-induced response, with transcript abundance increasing 6.76-fold at 24 h (Figure 6A).

During salt stress, all examined members of subgroups II and III were markedly upregulated. In particular, *TaPum-III-5b* showed an exceptionally strong response, with relative transcript abundance exceeding 10-fold at 24 h. In contrast, *TaPum-I-10a/b/d* were substantially repressed by NaCl treatment. Some genes, such as *TaPum-III-5b*, exhibited contrasting expression patterns under cold and salt stress, whereas *TaPum-II-1a/b/d* responded similarly to both treatments (Figure 6B). Among the nine qRT-PCR-validated candidates, *TaPum-III-5b* showed one of the strongest NaCl-induced responses, with transcript abundance exceeding 10-fold at 24 h, and exhibited a stress-response pattern distinct from that observed under cold treatment. This pronounced and salt-biased expression pattern prompted its selection for subsequent functional characterization.

### 2.6. TaPum-III-5b Overexpression in Arabidopsis Enhances Salt Tolerance

Based on its stress-responsive expression, we conducted further functional analysis on TaPum-III-5b. Subcellular localization analysis in epidermal cells of tobacco leaves revealed nuclear and cytoplasmic localization of TaPum-III-5b fused to green fluorescent protein (GFP), with 35S-GFP used as a positive control (Figure 7A).

Transgene-specific PCR screening identified 24 positive T0 lines. Subsequent qRT-PCR analysis showed that Over-2 and Over-6 had the highest *TaPum-III-5b* expression levels among these lines, with relative expression values of 17.72 and 14.92, respectively (Supplementary Figure S3); these two independently recovered lines were therefore selected for subsequent experiments. Both lines exhibited enhanced survival under salt stress. Over-2 showed a slightly higher survival rate than Over-6, consistent with its higher transgene expression level, indicating that the two-line dataset does not establish a quantitative correlation between *TaPum-III-5b* expression and phenotype severity.

To investigate its function under salt stress, we generated stable *Arabidopsis* lines overexpressing *TaPum-III-5b* and advanced them to the T3 generation. Two independent overexpression lines (Over-2 and Over-6) and wild-type (WT) Col-0 plants underwent NaCl treatment. Following salt stress, 16 of 27 Over-2 plants (59.26%) and 14 of 27 Over-6 plants (51.85%) survived, compared with 7 of 27 WT plants (25.92%) (Figure 7B,C). The surviving/initial plant numbers in the three replicate pots were 2/9, 2/9, and 3/9 for WT; 5/9, 5/9, and 6/9 for Over-2; and 4/9, 5/9, and 5/9 for Over-6.

Grouped binomial logistic regression detected a significant overall effect of genotype on survival (likelihood-ratio χ^2^ = 6.89, df = 2, *p* = 0.032). A planned contrast comparing the average effect of the two independent overexpression lines with WT showed significantly higher survival odds in the overexpression lines (odds ratio = 3.58, 95% confidence interval = 1.30–9.87, *p* = 0.014).

Nitroblue tetrazolium (NBT) and 3,3′-diaminobenzidine (DAB) histochemical staining showed lower superoxide radical (O_2_•^−^) and hydrogen peroxide (H_2_O_2_) accumulation, respectively, in overexpression line leaves under salt-stress conditions (Figure 7D). Physiological analyses revealed increased antioxidant enzyme activities following *TaPum-III-5b* overexpression during salt stress. Under salt stress, SOD, CAT, and POD activities in the overexpression lines were 1.25- to 1.27-, 1.09- to 1.15-, and 1.39- to 1.41-fold those of salt-treated WT plants, respectively. Malondialdehyde (MDA) and ROS levels were 15.33–22.42% and 42.46–45.46% lower, respectively, than those in salt-treated WT plants. Soluble sugar and soluble protein contents were 1.45-fold and 1.32- to 1.34-fold those of salt-treated WT plants, respectively, whereas proline content did not differ significantly between the overexpression lines and WT under salt stress (Figure 7E).

These results show that heterologous expression of *TaPum-III-5b* in *Arabidopsis* increased salt tolerance and was associated with reduced oxidative damage and improved osmotic adjustment.

### 2.7. TaPum-III-5b Is Transcriptionally Activated by TaDREB2B

To determine whether *TaPum-III-5b* overexpression was associated with changes in salt-responsive gene expression, the transcript levels of several salt-responsive genes were examined in *Arabidopsis*. These genes showed little variation in transcript abundance among genotypes under control conditions. Following NaCl treatment, however, genes involved in ion transport (*AtSOS1*, *AtNHX1*, and *AtHKT1*), proline biosynthesis (*AtP5CS1*), and stress-responsive transcriptional regulation (*AtDREB2A* and *AtNAC019*) were strongly induced in all lines. This induction was substantially greater in the overexpressing lines Over-2 and Over-6 compared to WT plants (Figure 8A). These expression changes represent downstream responses associated with *TaPum-III-5b* overexpression but do not demonstrate direct post-transcriptional regulation of these transcripts.

We subsequently investigated the upstream transcriptional regulators of *TaPum-III-5b*. Analysis of its promoter identified multiple dehydration-responsive elements (DREs), which are canonical binding sites for AP2/ERF-type dehydration-responsive element-binding (DREB) TFs. Screening of the UniProt database (accessed 21 December 2025) for wheat DREB proteins associated with salt stress identified TaDREB2B as the most relevant candidate [34]. qRT-PCR analysis showed that *TaDREB2B* expression in leaves of Luyan951 seedlings was significantly induced at 6, 12, and 24 h after treatment with 200 mM NaCl and reached its highest level at 24 h (Figure 8B). Based on this salt-responsive expression pattern and the presence of DRE motifs in the *TaPum-III-5b* promoter, TaDREB2B was examined as a candidate upstream regulator. An electrophoretic mobility shift assay (EMSA) showed that recombinant maltose-binding protein (MBP)-TaDREB2B bound directly to the 20-bp wild-type (WT) probe derived from the *TaPum-III-5b* promoter. The shifted complex was progressively reduced by 10- and 50-fold molar excesses of unlabeled WT competitor, whereas no shifted complex was observed with the 5′-biotin-labeled mutant probe, supporting sequence-specific binding (Figure 8C).

The dual-luciferase (Dual-LUC) assay further showed that TaDREB2B significantly activated the wild-type *TaPum-III-5b* promoter in rice protoplasts relative to the empty-vector control (Figure 8D). Together with the EMSA results, these data demonstrate in vitro binding of TaDREB2B to the DRE-containing promoter sequence and activation of the wild-type promoter. However, because a DRE-mutated promoter reporter was not examined in the Dual-LUC assay, the dependence of promoter activation on the DRE motif remains to be determined.

## 3. Discussion

RBPs contribute to the development, growth, and stress responses of plants [35,36]. Proteins from the Pum family carry a conserved Pum-HD that recognizes sequences within the 3′-UTR of target mRNAs and regulates their translation and stability [37]. Although several studies have examined Pum proteins in animals and fungi, their functions in plants, particularly crop species, remain unclear [17]. Here, we characterized the wheat *TaPum* gene family using genome-wide analyses. Our results associate the *TaPum* family with salt-stress responses and show that heterologous expression of *TaPum-III-5b* enhances salt tolerance in *Arabidopsis*, while the EMSA and Dual-LUC assays support TaDREB2B as a candidate upstream regulator.

The wheat *TaPum* family comprises 56 members, but this number must be interpreted in the context of the allohexaploid wheat genome. Per subgenome, wheat contains approximately 18.7 *TaPum* genes, which is close to the number in maize (19) and lower than that in *Arabidopsis* (26) [19]. According to synteny analysis, the current *TaPum* family size reflects A/B/D homoeologous retention together with segmental and tandem duplication. After separating homoeologous pairs, 17 non-homoeologous segmental duplication pairs and five tandem duplication pairs remained; all duplicated gene pairs showed Ka/Ks ratios below 1, consistent with purifying selection after duplication. Nevertheless, subgroup-specific auxiliary domains and the uneven distribution of *cis*-acting elements suggest potential regulatory divergence following gene duplication (Figure 2; Figure 4). The most abundant hormone-responsive elements in *TaPum* promoters were ABA- and MeJA-responsive elements. MeJA-responsive elements were particularly enriched in subgroup III, with *TaPum-III-1b* containing 16 such elements, compared with 0 to 6 in most members of subgroups I and II. This distribution suggests that subgroup III may be associated with a greater abundance of predicted jasmonate-responsive promoter elements. However, functional jasmonic acid (JA) or ABA responsiveness cannot be inferred from these predictions alone, although both hormones are broadly involved in abiotic stress responses [38,39]. In *Arabidopsis*, *AtPUM23* has also been linked to ABA biosynthesis and signaling under salt stress [40]. Its mutation results in hypersensitivity to salt that can be rescued by exogenous ABA, providing biological context for the prediction without validating the function of these elements in wheat *TaPum* promoters.

Based on transcriptomic and qRT-PCR analyses, *TaPum* genes demonstrated a more consistent and pronounced response to salt stress than to cold or drought stress (Figure 5; Figure 6). Heterologous expression of *TaPum-III-5b* in *Arabidopsis* enhanced salt tolerance, as indicated by increased survival and reduced cellular damage (Figure 7). The enhanced salt tolerance of the overexpression lines was accompanied by higher SOD, CAT, and POD activities, reduced ROS and MDA accumulation, and increased soluble sugar and soluble protein contents. These physiological changes are consistent with an improved antioxidant and osmotic-adjustment response under salt stress [41,42,43]. However, the present data do not establish that TaPum-III-5b directly regulates antioxidant genes or enzymes, and its downstream post-transcriptional targets require further investigation. AtPUM5 has similarly been implicated in salt-stress responses through its interaction with the 3′-UTR of *RD22*, illustrating the diverse functions of Pum proteins in stress regulation [22].

Subcellular localization analysis showed nuclear and cytoplasmic localization of the TaPum-III-5b–GFP fusion protein (Figure 7A). This distribution is consistent with that of many RBPs that shuttle between these compartments and participate in processes such as precursor messenger RNA (pre-mRNA) splicing, nuclear export, and cytoplasmic translational regulation [16,44]. Dual-localization may therefore allow TaPum-III-5b to regulate RNA metabolism at multiple stages. In contrast, AtPUM6 is anchored to the endoplasmic reticulum surface and functions as a post-translational regulator, and its silencing enhances salt tolerance [45]. These contrasting localization patterns further suggest that the functions of Pum proteins in stress adaptation are influenced by their subcellular distributions.

To investigate upstream regulation of *TaPum-III-5b*-mediated salt tolerance, we identified TaDREB2B as a candidate upstream regulator. DREB TFs are AP2/ERF superfamily members and are major regulators of plant responses to abiotic stresses, including salinity [46,47]. EMSA demonstrated sequence-specific binding of TaDREB2B to the DRE-containing *TaPum-III-5b promoter* sequence in vitro, whereas the Dual-LUC assay showed that TaDREB2B transactivated the wild-type promoter in rice protoplasts. Whether this transcriptional activation is specifically dependent on the DRE motif remains to be verified using a DRE-mutated promoter reporter. Direct occupancy of the endogenous *TaPum-III-5b promoter* in wheat remains to be established by chromatin immunoprecipitation followed by quantitative PCR (ChIP-qPCR) or an equivalent assay. Together, these findings support TaDREB2B as a candidate upstream regulator of *TaPum-III-5b* rather than demonstrating endogenous promoter occupancy in wheat. Given the established functions of Pumilio proteins as RNA-binding proteins, *TaPum-III-5b* may participate in downstream post-transcriptional regulation, but this possibility remains to be tested. The increased expression of genes such as *AtSOS1* and *AtNHX1*, which contribute to ion homeostasis, in the TaPum-III-5b-overexpressing lines is consistent with a role for *TaPum-III-5b* in the broader salt response network [48,49,50]. However, these expression changes do not establish that these transcripts are direct targets of TaPum-III-5b.

This study has two main limitations. First, the functional analysis of *TaPum-III-5b* was based solely on heterologous expression in *Arabidopsis*, whereas the polyploid wheat genome may involve additional regulatory complexity. Functional validation through stable transformation and clustered regularly interspaced short palindromic repeats (CRISPR)/CRISPR-associated protein 9 (Cas9)-mediated knockout in wheat will therefore be needed. Second, the direct RNA targets of TaPum-III-5b remain unknown. Future RNA immunoprecipitation sequencing analyses will be needed to identify its bound transcripts and clarify the post-transcriptional regulatory network underlying salt tolerance in wheat.

## 4. Materials and Methods

### 4.1. TaPum Gene Family Member Identification

Genome sequences and annotation files for *Arabidopsis* (The *Arabidopsis* Information Resource 10 (TAIR10); https://www.arabidopsis.org/download/list?dir=Sequences%2FTAIR10_blastsets; date of access: 20 September 2024), maize (B73 v5), and wheat (International Wheat Genome Sequencing Consortium (IWGSC) RefSeq v1.0 and RefSeq Annotation v1.1) were accessed from the Ensembl Plants database (https://plants.ensembl.org/Triticum_aestivum/Info/Index; date of access: 20 September 2024). The Pfam database was used to obtain the hidden Markov model profile of the Pum-HD (PF00806) (http://pfam.xfam.org/; date of access: 20 September 2024); candidate Pum proteins were identified using hidden Markov model search software (HMMER) v3.4 with an E-value threshold of 1 × 10^−5^ [51]. Local Basic Local Alignment Search Tool for proteins (BLASTP) searches were also performed using AtPUM protein sequences as queries to identify TaPum proteins, using an E-value threshold of 1 × 10^−5^; hits with both sequence identity and query coverage > 80% were retained as candidate genes [19]. When multiple protein isoforms were annotated for the same gene locus, only the longest protein isoform was retained. Candidate sequences independently recovered by HMMER and BLASTP were merged according to their stable gene identifiers, and duplicate identifiers and identical protein sequences were removed. The presence and integrity of the Pum-HD were subsequently verified using Pfam (PF00806), the National Center for Biotechnology Information Conserved Domain Database (NCBI CDD), and the Simple Modular Architecture Research Tool (SMART); sequences lacking a complete Pum-HD were excluded from further analysis. Expert Protein Analysis System (ExPASy) (http://expasy.org/tools/; date of access: 7 October 2024) was used to analyze the identified TaPum proteins’ physicochemical properties [52]. SWISS-MODEL (https://swissmodel.expasy.org/; date of access: 11 September 2026) was used to predict protein structures (ProMod3 v3.6.0; SWISS-MODEL Template Library (SMTL) release 10 September 2026). One representative protein was selected from each phylogenetic group and species, with preference given to complete sequences and models with higher coverage and quality scores. The selected protein identifiers, templates, sequence identities, coverage, Global Model Quality Estimate (GMQE) values, mean local model confidence scores, and Pum-HD/PUF-family Pfam match positions are provided in Supplementary Table S6.

### 4.2. Phylogenetic, Gene Structure, Conserved Motif, Conserved Domain, and Cis-Acting Element Analyses

Protein sequences of Pum family members identified from wheat, maize, and *Arabidopsis* were obtained from the Ensembl Plants database and imported into Molecular Evolutionary Genetics Analysis version X (MEGA-X). Protein sequences were aligned using Multiple Sequence Comparison by Log-Expectation (MUSCLE) in MEGA-X with the default alignment parameters. The resulting alignment comprised 101 protein sequences and 1503 amino-acid positions. No manual or automated post-alignment trimming was applied. The best-fitting amino-acid substitution model was evaluated using the Find Best DNA/Protein Models (ML) procedure in MEGA-X, and the model with the lowest Bayesian information criterion was selected. The Jones–Taylor–Thornton (JTT) model with uniform rates among sites was used for maximum-likelihood tree reconstruction. Sites containing gaps or missing data were treated using partial deletion with a 95% site-coverage cutoff, retaining 272 positions for phylogenetic analysis. The unrooted maximum-likelihood tree was assessed using 1000 bootstrap replicates. The underlying protein alignment and maximum-likelihood tree are provided as Supplementary Data S1 and Supplementary Data S2, respectively. The Interactive Tree of Life (https://itol.embl.de/; date of access: 1 January 2025) was used to visualize the resultant tree.

Batch Conserved Domain Search (CD-Search) was used for detecting TaPum proteins’ conserved domains [53]. The Multiple EM for Motif Elicitation (MEME) online server (https://meme-suite.org/meme/; date of access: 27 October 2024) was used to identify conserved motifs, with 15 as the maximum motif number [54]. Gene structures, including UTRs, exons, and introns, were characterized using the General Feature Format (GFF) annotation files obtained from Ensembl Plants. To analyze *cis*-acting elements, the genomic sequence (2000 bp) immediately upstream of each *TaPum* gene’s translation initiation codon (ATG) was screened by PlantCARE (https://bio.tools/plantcare; date of access: 26 October 2024) [55]. All results were visualized using TBtools-II v2.487 [56].

### 4.3. Chromosomal Distribution, Collinearity, Ka/Ks, and Gene Expression Analyses

*TaPum* genes’ chromosomal positions were determined according to their physical coordinates in the wheat genome annotation obtained from Ensembl Plants. Chromosome length information from the wheat GFF annotation file was used to map the density and distribution of *TaPum* genes across chromosomes. Chromosomal localization was identified by TBtools software. Gene duplication patterns and collinearity among *TaPum* genes were analyzed using MCScanX (v1.0.0) as implemented in TBtools. All 56 *TaPum* genes were assessed. A–B, A–D, and B–D collinear pairs were annotated as homoeologous pairs derived from polyploidization. After excluding these homoeologous pairs, non-homoeologous collinear pairs were classified as segmental duplication pairs. Tandem duplication pairs were defined as homologous genes located on the same chromosome and separated by no more than 10 intervening genes.

Wheat transcriptome datasets were obtained from WheatOmics 1.0 (https://wheatomics.sdau.edu.cn/expression/; date of access: 12 January 2025). The datasets comprised the Chinese Spring developmental transcriptome (PRJEB5314), the leaf low-temperature transcriptome (PRJNA253535/GSE58805), the drought, heat, and combined drought-heat transcriptome of one-week-old TAM 107 seedling leaves sampled at 0, 1, and 6 h (PRJNA257938/SRP045409), and the salt-stress transcriptome of Chinese Spring roots sampled after treatment with 150 mM NaCl for 6, 12, and 24 h, together with the corresponding controls (PRJNA293629). The drought and heat dataset contained two biological replicates per condition, whereas the low-temperature dataset contained three biological replicates per condition. Transcripts per million (TPM) values supplied by WheatOmics were averaged across the available biological replicates and transformed as log2(TPM + 1) for heatmap visualization.

### 4.4. Plant Materials, Growth Conditions, and Stress Treatments

The wheat cultivar Luyan951 was developed by the Crop Research Institute, Shandong Academy of Agricultural Sciences. Two-leaf and one-heart stage seedlings were exposed to NaCl treatment (200 mM) or low temperature (4 °C) for 24 h. Cultivation was performed under a 14-h light/10-h dark photoperiod in a greenhouse. At 0, 6, 12, and 24 h, leaves were collected, rapidly frozen in liquid nitrogen, and subsequently stored at −80 °C until analysis.

For generating the overexpression construct, the coding sequence (CDS) of *TaPum-III-5b* was inserted into the pC1300S-GFP vector between the *Kpn*I and *Bam*HI restriction sites (5ʹ-end and 3ʹ-end, respectively) (Supplementary Table S5). The floral dip method was used to transform *Arabidopsis* Col-0 plants with *Agrobacterium tumefaciens* LBA4404 carrying the cauliflower mosaic virus (CaMV) 35S-driven *TaPum-III-5b* construct [57]. Subsequent analyses were conducted with homozygous T_3_ transgenic lines. The stable transgenic lines expressed TaPum-III-5b as a GFP fusion protein.

Twenty-four independently recovered T0 transformants were screened using transgene-specific PCR, and the PCR-positive plants were subsequently analyzed for *TaPum-III-5b* expression by qRT-PCR (Supplementary Figure S3). Over-2 and Over-6 originated from different T0 transformants. Their T1 progenies showed an approximately 3:1 segregation ratio of transgene-positive to transgene-negative plants, consistent with single-locus Mendelian inheritance. Homozygous progenies were selected and advanced to the T3 generation for subsequent experiments.

*Arabidopsis* seeds were stratified at 4 °C in darkness for 3 days and germinated on half-strength Murashige and Skoog (MS) medium for 5–6 days. Uniform seedlings were transplanted into 10 × 10 cm pots containing a 1:1 (*v*/*v*) mixture of vermiculite and nutrient soil, with nine plants per pot. Each genotype comprised three biological replicates, with one pot per replicate. Plants were grown for 3 weeks under a 14-h light/10-h dark photoperiod, day/night temperatures of 22/20 °C, 60–70% relative humidity, and a light intensity of approximately 120 μmol m^−2^ s^−1^. Col-0 and transgenic plants were then irrigated with 1 L of 200 mM NaCl every 3 days for 3 weeks. Pots were repositioned daily to minimize positional effects. At the end of treatment, plants retaining green, turgid rosette leaves and an upright shoot were scored as surviving, whereas plants with fully chlorotic or necrotic, collapsed rosettes were scored as dead. Plants were assigned to pot positions at random before treatment. Survival was scored by an investigator blinded to genotype identity.

### 4.5. RNA Extraction and qRT-PCR

The RNAprep Pure Plant Plus Kit (DP441; Tiangen, Beijing, China) was used to extract total RNA from wheat leaves. The First-Strand cDNA Synthesis Kit (Vazyme, Nanjing, China) was used to synthesize first-strand cDNA. qRT-PCR was conducted using a Roche LightCycler 480 II (Roche Diagnostics, Mannheim, Germany) system coupled with SYBR Green-based Super Real PreMix Plus (Tiangen, Beijing, China). SnapGene v6.0 was used for constructing gene-specific primers; these primers were validated using NCBI Primer-BLAST (https://www.ncbi.nlm.nih.gov/tools/primer-blast/; date of access: 27 October 2024). Supplementary Table S5 lists the primer sequences. We selected the *ACTIN* gene for internal reference because its expression showed no or only slight variation among treatments (|ΔCt| ≤ 0.5). The 2^−ΔΔCt^ method was used to estimate relative transcript levels. For each treatment and genotype, three independently collected biological samples were analyzed, and each biological sample was measured using three technical reactions. Technical reactions were averaged within each biological replicate and were not treated as independent replicates [58].

### 4.6. Measurement of Physiological Parameters and ROS Metabolism

At the end of the 3-week NaCl treatment, fully expanded rosette leaves were collected from Col-0, Over-2, and Over-6 plants and immediately used for all biochemical assays and histochemical staining. For each genotype and treatment, three independently collected biological samples were analyzed, with each biological replicate consisting of leaf tissue collected independently from one replicate pot. Antioxidant enzyme activities and osmolyte, soluble protein, and ROS contents were measured with commercial assay kits (Solarbio, Beijing, China). The caffeic acid colorimetric method (BC0090), NBT colorimetric method (BC5160), and ammonium molybdate colorimetric method (BC0200) were used to quantify POD, SOD, and CAT activities, respectively. MDA, proline, and soluble sugar contents were estimated with the thiobarbituric acid method (BC0300), ninhydrin colorimetric method (BC0290), and anthrone colorimetric method (BC0030), respectively. Soluble protein and ROS levels were measured by the Coomassie Brilliant Blue method (BC0040) and titanium sulfate colorimetric method (BC3590), respectively. Histochemical staining using DAB and NBT was performed following previously described protocols [59].

### 4.7. Subcellular Localization

After introduction into *A. tumefaciens* EHA105, the fusion construct was transiently expressed in the leaves of *Nicotiana tabacum* by agroinfiltration. After 48 h, fluorescence signals were captured with a laser-scanning confocal microscope at 488 nm excitation wavelength. Supplementary Table S5 lists the primer sequences.

### 4.8. Dual-Luciferase Reporter Assay

An approximately 2-kb TaPum-III-5b promoter fragment was cloned into the pGreenII 0800-LUC reporter vector, which contains firefly luciferase (LUC) and Renilla luciferase (REN) as the internal control, and the *TaDREB2B* CDS was cloned into the pGreenII 62-SK effector vector. The reporter was co-transfected into rice protoplasts with either TaDREB2B-pGreenII 62-SK or empty pGreenII 62-SK at a 1:1 molar ratio using polyethylene glycol (PEG)-mediated transformation. After 20 h of incubation in darkness, LUC and REN activities were measured using a dual-luciferase reporter assay kit (Vazyme, Nanjing, China), and promoter activity was normalized as the LUC/REN ratio. Three independent protoplast transfections were performed, with each transfection treated as one biological replicate; the LUC/REN ratios were compared using Student’s *t*-test. Primer sequences are listed in Supplementary Table S5.

### 4.9. EMSA

Maltose-binding protein (MBP)-TaDREB2B was expressed and purified using the pMAL-C5X vector. Each binding reaction contained 1 μL of purified MBP-TaDREB2B at 0.28 μg μL^−1^ (0.28 μg total) and a 1 μM 5′-biotin-labeled 20-bp wild-type (WT) probe (5′-ACAAGCAACCGACGAGGATG-3′; reverse complement, 5′-CATCCTCGTCGGTTGCTTGT-3′) located at positions 115–134 of the 2-kb *TaPum-III-5b* promoter fragment, corresponding to −1886 to −1867 bp relative to the translational start site when the promoter is numbered from −2000 to −1 bp. Competition reactions contained 10- or 50-fold molar excesses of unlabeled WT probe. A 5′-biotin-labeled mutant probe (5′-ACAAGCATTTTTTGAGGATG-3′; reverse complement, 5′-CATCCTCAAAAAATGCTTGT-3′) was used to test sequence specificity. After incubation at 4 °C for 3 min, complexes were separated on 6% native polyacrylamide gels and detected using a chemiluminescent electrophoretic mobility shift assay (EMSA) kit (Thermo Fisher Scientific, Rockford, IL, USA) and a ChemiDoc MP (Bio-Rad, Hercules, CA, USA) imaging system. Probe sequences are listed in Supplementary Table S5.

### 4.10. Statistical Analysis

Survival and other quantitative measurements were analyzed separately. For survival analysis, the surviving and dead plant counts in each pot were analyzed using a grouped binomial generalized linear model with a logit link. Genotype was included as a fixed factor, and each pot was treated as an independent experimental unit. Model dispersion was assessed using the Pearson χ^2^ statistic divided by the residual degrees of freedom. The overall genotype effect was evaluated using a likelihood-ratio test. A planned contrast comparing the average effect of the two independent overexpression lines with WT was evaluated using a two-sided Wald test. Physiological and biochemical data were analyzed using two-way ANOVA with genotype and treatment as fixed factors, including the genotype × treatment interaction, followed by the Dunnett multiple-comparison test comparing each overexpression line with WT within the same treatment. Data are presented as the mean ± SD of three independent biological replicates. Technical measurements were averaged within each biological replicate and were not treated as independent observations. A value of *p* < 0.05 was considered statistically significant.

## 5. Conclusions

We characterized the wheat *TaPum* gene family. The 56 identified *TaPum* genes exhibited diverse structural features, evolutionary conservation, and distinct patterns of expression in various tissues, developmental stages, and abiotic stress conditions. A total of 25 A/B/D homoeologous pairs, 17 segmental duplication pairs, and five tandem duplication pairs were identified. The larger total family size in wheat is partly explained by its allohexaploid genome, and both homoeologous retention and segmental/tandem duplication contributed to the current *TaPum* family size. The coordinated induction of many family members under salt stress indicated their potential involvement in salinity responses. Functional analysis showed that heterologous expression of *TaPum-III-5b* in *Arabidopsis* enhanced salt tolerance and was accompanied by increased antioxidant enzyme activities, reduced oxidative damage, and improved osmotic adjustment. EMSA showed that TaDREB2B binds the *TaPum-III-5b* promoter sequence in vitro, whereas Dual-LUC analysis showed transactivation of the wild-type promoter in rice protoplasts. These findings position the TaDREB2B-*TaPum-III-5b* relationship within a proposed regulatory framework associated with salt-stress responses. Further validation of endogenous promoter occupancy and *TaPum-III-5b* function in wheat is required before its potential application in wheat improvement can be established.

## Figures and Tables

**Figure 1:**
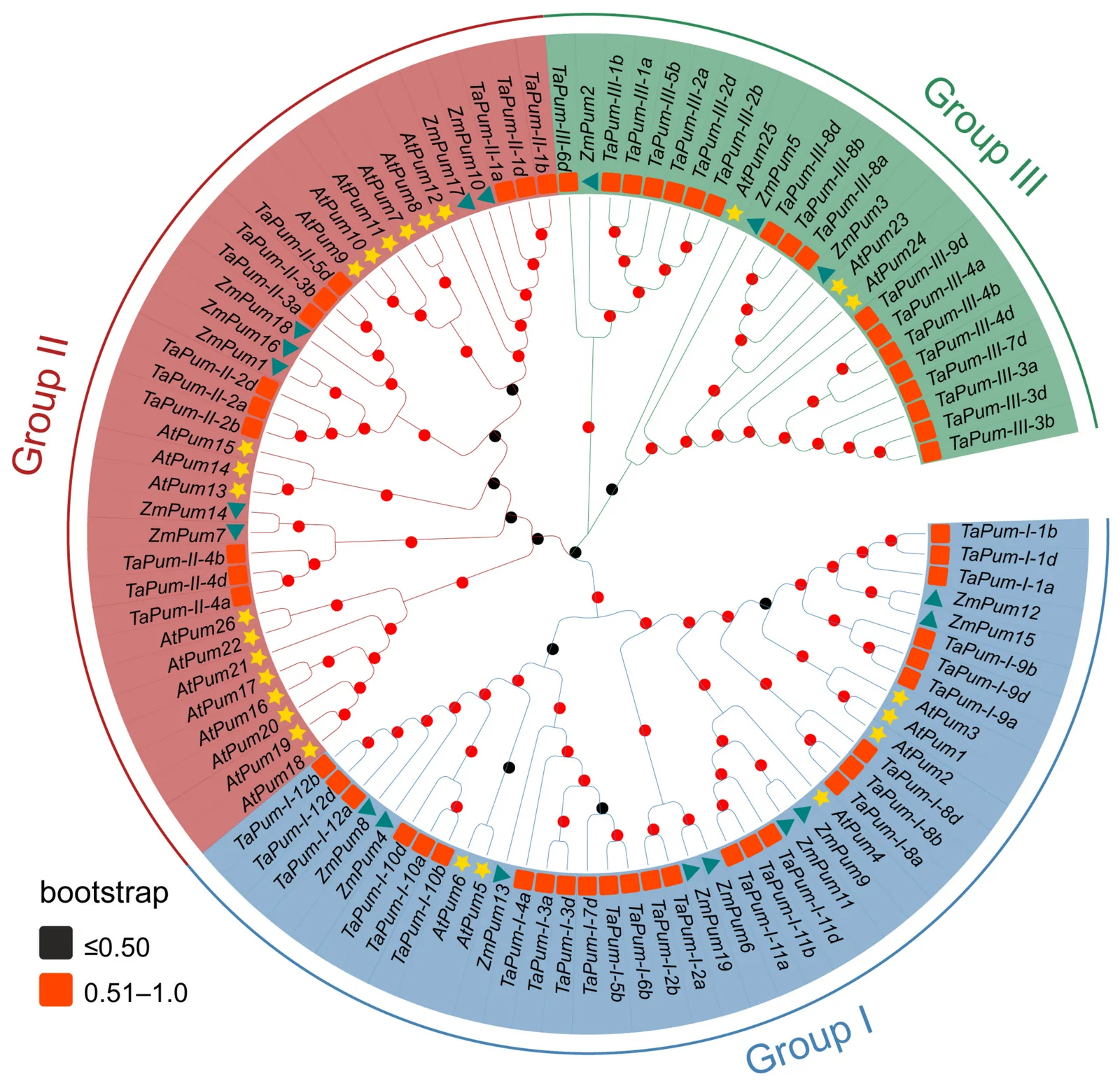
Phylogenetic study of Pum-family proteins from wheat (Ta), *Arabidopsis thaliana* (At), and maize (Zm). Pum proteins from *Arabidopsis*, wheat, and maize are shown as yellow stars, orange squares, and green triangles, respectively. Node colors indicate bootstrap support: gray, values ≤ 0.5; orange, values > 0.5 and <1. Values ≤ 0.50 indicate weakly supported relationships and were not used to infer detailed branching order.

**Figure 2:**
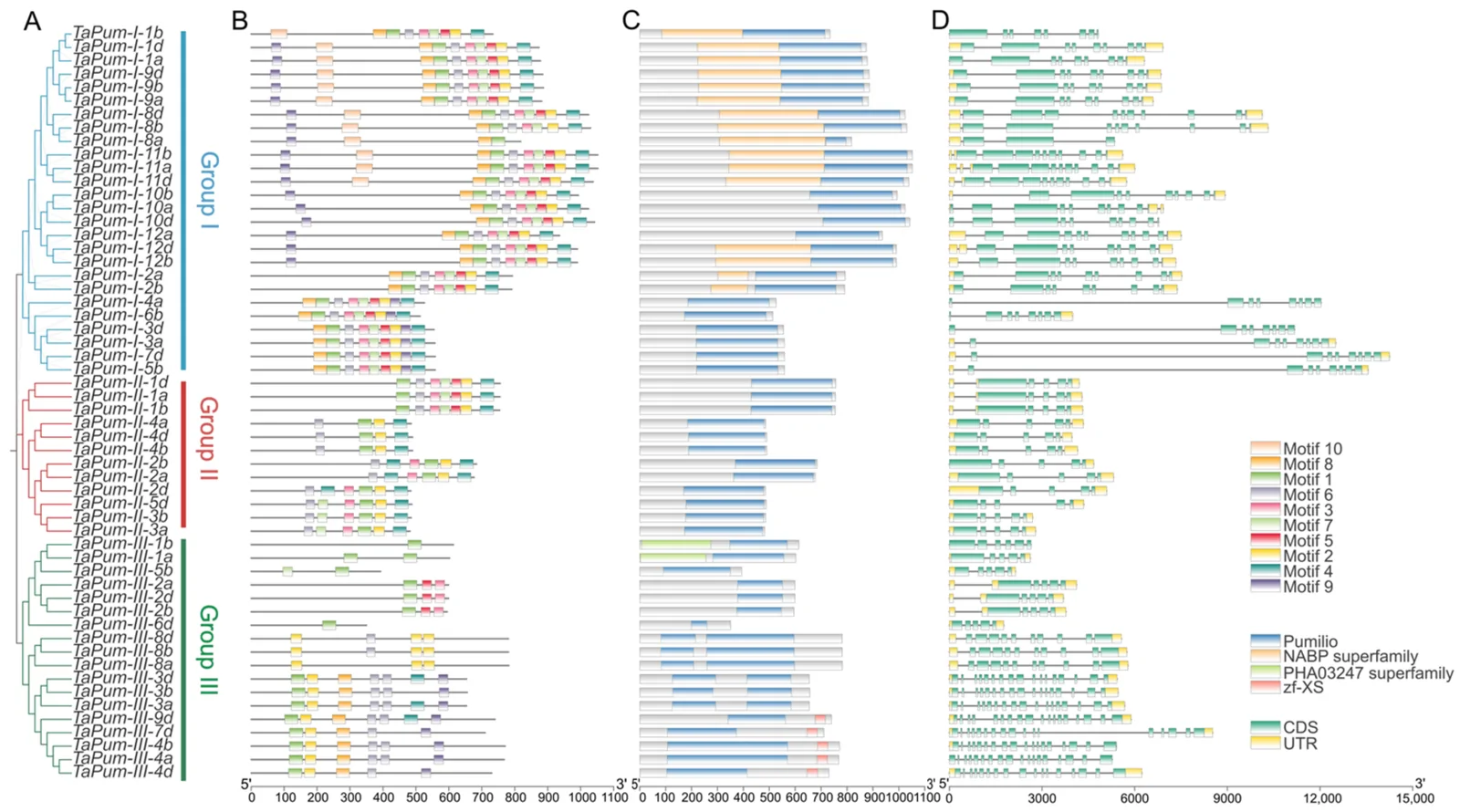
Characterization of the wheat Pum family with regard to gene structures, conserved domains and motifs, and phylogenetic relationships. (**A**) Phylogenetic tree of *TaPum* genes. (**B**,**C**) Conserved motif composition and conserved domain architecture of TaPum proteins, respectively. (**D**) Details of exon–intron structures of *TaPum* genes.

**Figure 3:**
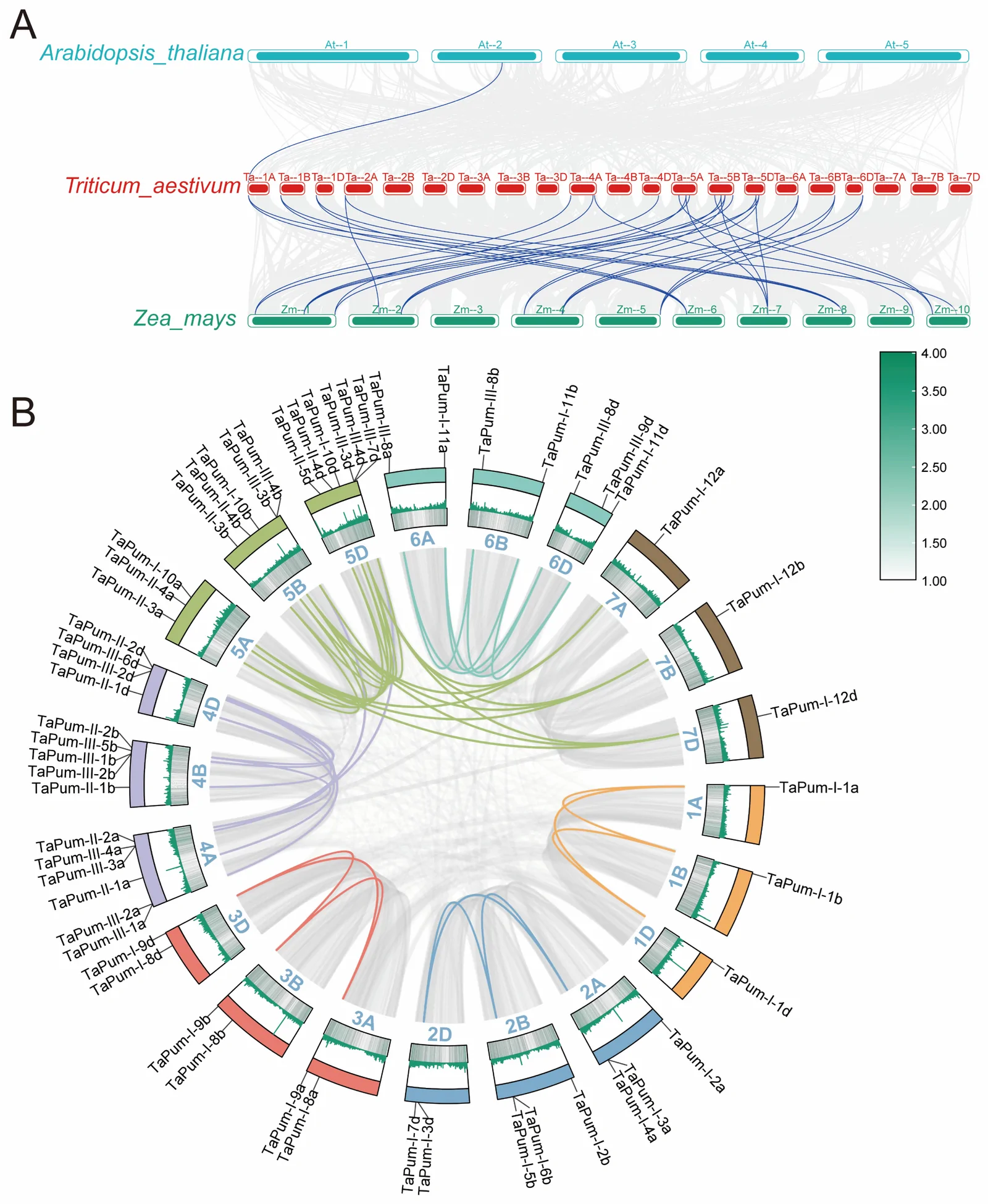
Collinearity analysis of *TaPum*-family genes in wheat. (**A**) Syntenic relationships of Pum genes among common wheat, *Arabidopsis thaliana*, and maize. In the background, grey lines denote syntenic blocks within the wheat genome and between genomes of wheat and other plants; blue lines link syntenic gene pairs. (**B**) Chromosomal distribution as well as synteny analysis of wheat *TaPum* genes. Syntenic gene pairs are indicated by colored arcs, different colors distinguish A/B/D homoeologous pairs, segmental duplication pairs, and tandem duplication pairs. The two outermost rings denote gene density along the chromosomes.

**Figure 4:**
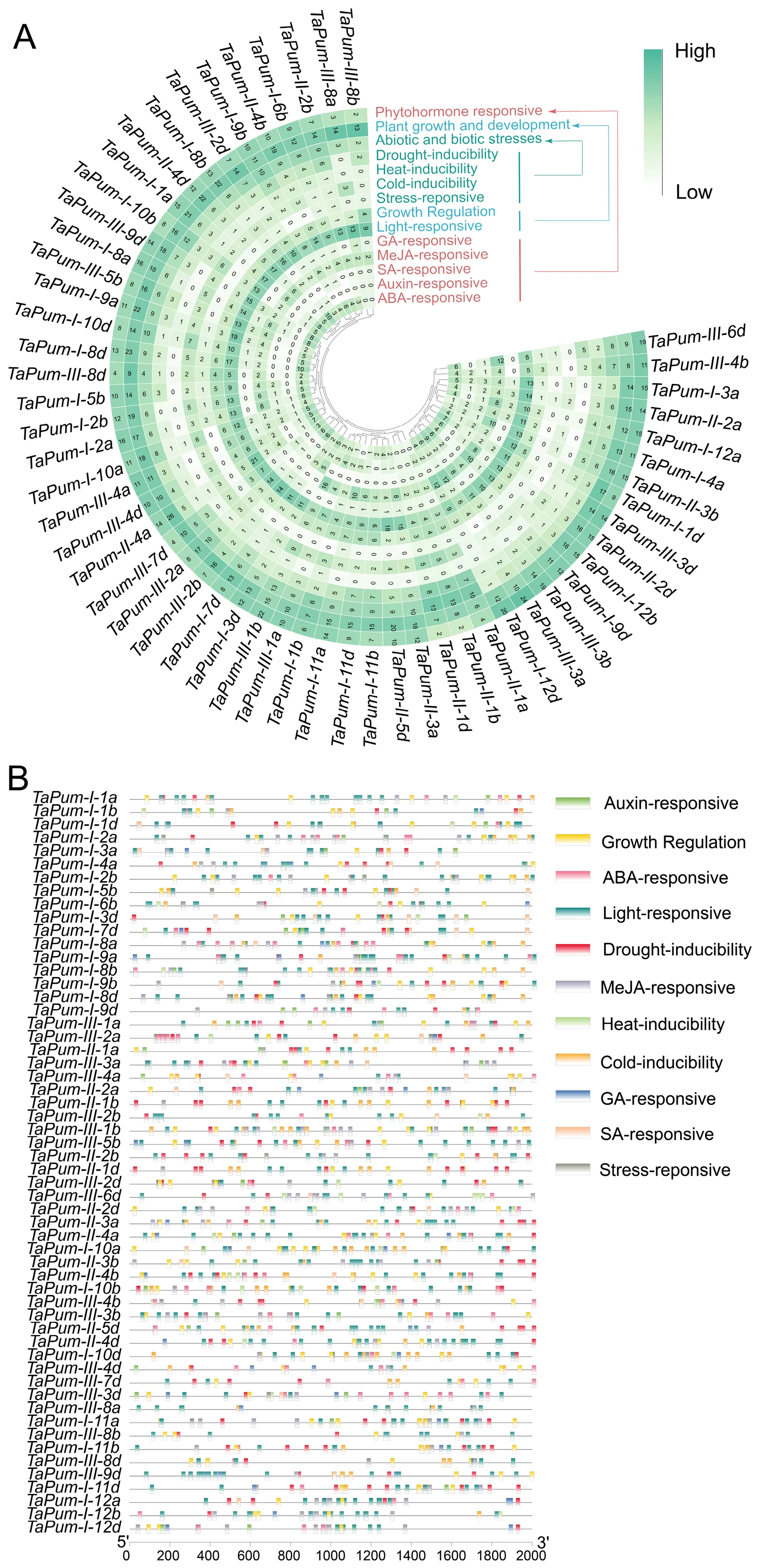
*cis*-acting regulatory elements predicted in wheat *TaPum* genes. (**A**) Abundance of the 11 classes of *cis*-acting regulatory elements in each *TaPum* promoter. (**B**) Types and distribution of *cis*-acting regulatory elements in *TaPum* promoters. Various *cis*-acting regulatory elements are shown by colored boxes; their names are provided on the right side.

**Figure 5:**
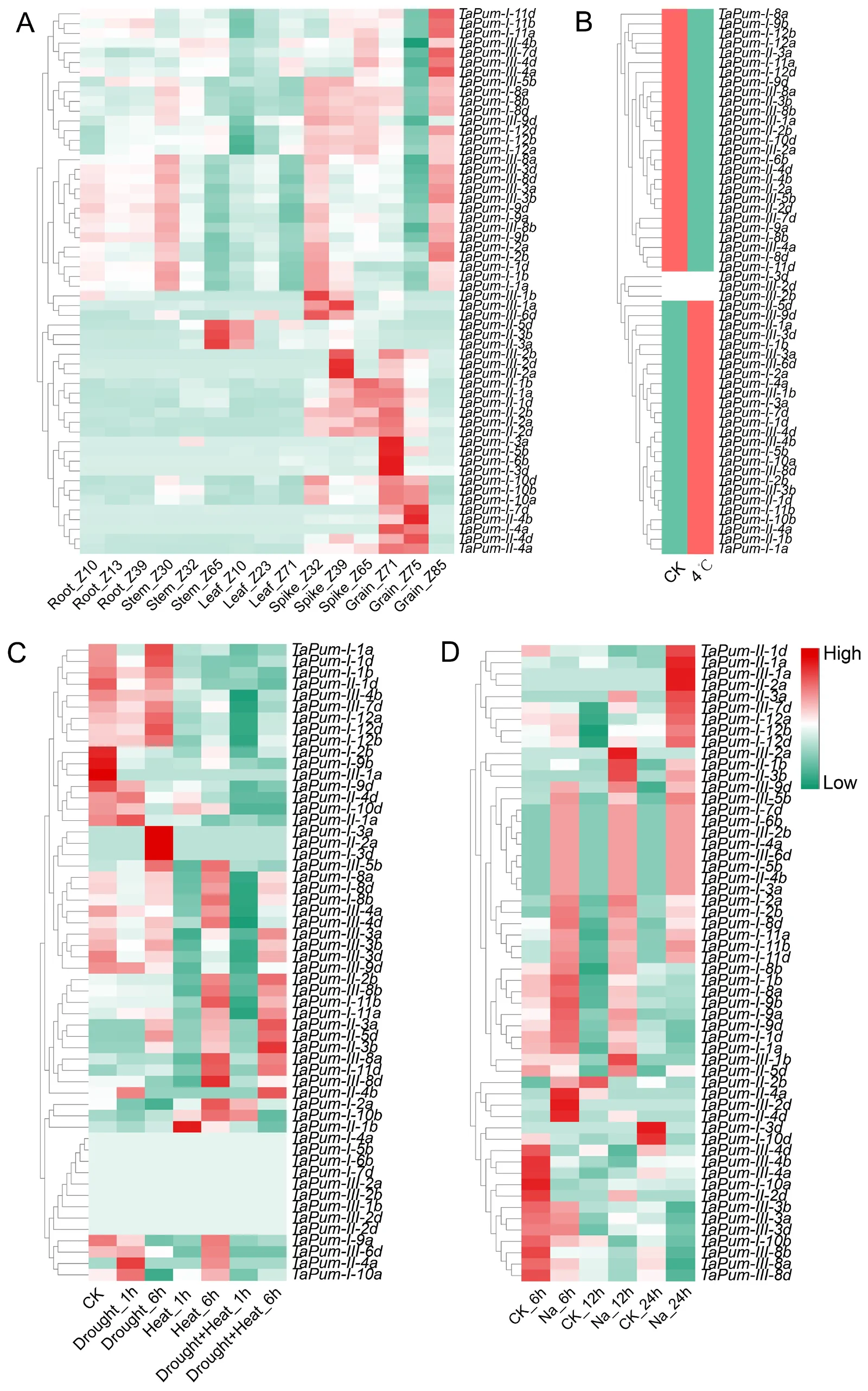
*TaPum* gene expression profile in wheat. (**A**) Expression patterns in tissues at various developmental stages in vegetative and reproductive organs. (**B**) Expression changes under cold stress (4 °C) relative to the control (CK). (**C**) Time-course expression after exposure to heat, drought, and combined drought–heat stress for 1 and 6 h. (**D**) Expression dynamics after exposure to salt stress for 6, 12, and 24 h. Hierarchical clustering dendrograms are shown on the left. The colour scale represents log2-transformed transcripts per million [log2(TPM + 1)] values; red and green indicate relatively high and low expression, respectively.

**Figure 6:**
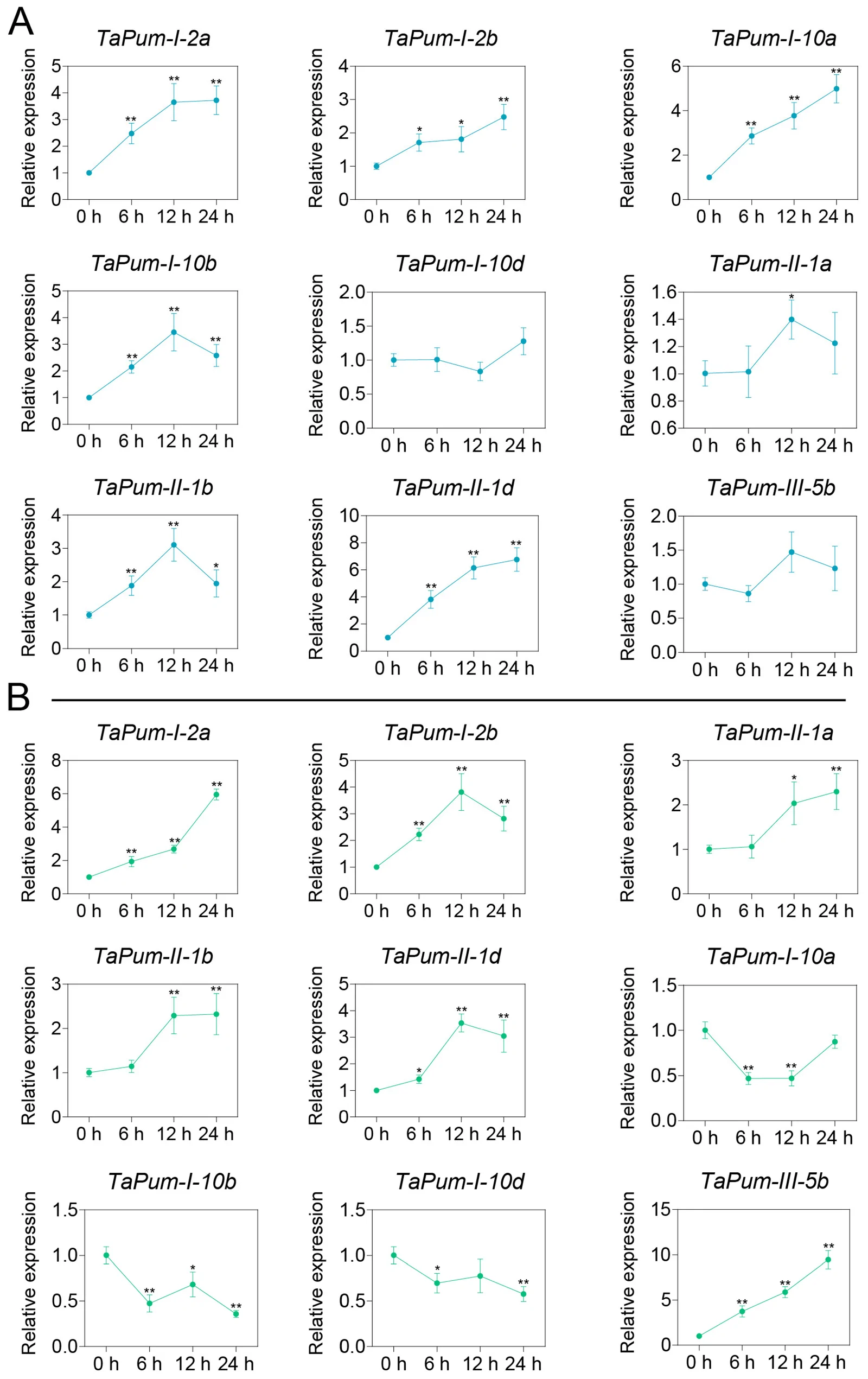
Quantitative reverse transcription polymerase chain reaction (qRT-PCR) analysis of nine selected *TaPum* genes exposed to cold and salt stress. (**A**) Relative transcript levels after cold treatment for 6, 12, and 24 h. (**B**) Relative transcript levels after salt treatment for 6, 12, and 24 h. Two-way analysis of variance (ANOVA) was used for evaluating statistical significance: * *p* < 0.05, ** *p* < 0.01.

**Figure 7:**
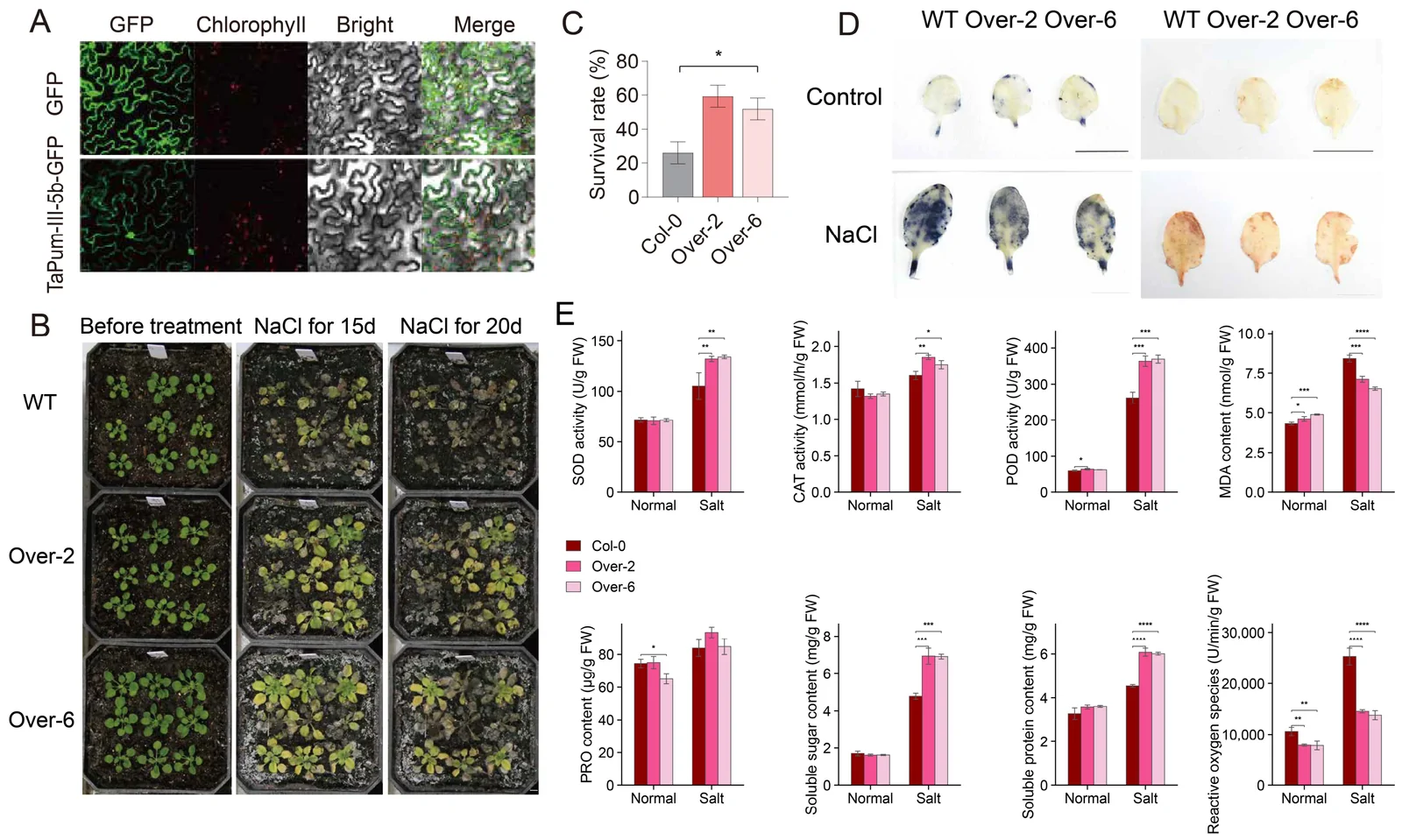
Salt tolerance analysis of *Arabidopsis thaliana* plants heterologously overexpressing *TaPum-III-5b*. (**A**) Subcellular localization of *TaPum-III-5b*. Chlorophyll, chlorophyll autofluorescence; GFP, green fluorescent protein; Bright, bright-field image; Merge, overlaid image. Scale bars are shown in the panels. (**B**) Phenotypes of WT (Col-0/WT) and *TaPum-III-5b*-overexpressing lines (Over-2 and Over-6) under NaCl treatment. (**C**) Survival rates of Col-0, Over-2, and Over-6 seedlings under salt stress. Survival data are presented as the mean ± standard deviation (SD) of three replicate pots, with nine plants per pot. The surviving and dead plant counts in each pot were analyzed using a grouped binomial generalized linear model with a logit link, with genotype as a fixed factor and each pot as an independent experimental unit. The overall genotype effect was evaluated using a likelihood-ratio test, and a planned contrast compared the average effect of the two independent overexpression lines with WT. The bracket indicates this contrast (* *p* = 0.014). (**D**) Histochemical detection of ROS. NBT (blue) and DAB (red) staining was performed to detect superoxide anion and hydrogen peroxide, respectively. Leaves were collected from plants exposed to control and NaCl-treated conditions. (**E**) Quantitative analysis of biochemical and physiological indices under control (normal) and salt-stress conditions, including contents of soluble proteins, proline (PRO), soluble sugars, MDA, and total ROS and catalase (CAT), superoxide dismutase (SOD), and peroxidase (POD) activities. Data are presented as the mean ± SD of three independent biological replicates. Statistical significance was assessed using two-way ANOVA with genotype and treatment as fixed factors, including their interaction, followed by the Dunnett multiple-comparison test. Brackets connect each overexpression line with WT within the same treatment, and asterisks indicate the corresponding significance levels: * *p* < 0.05, ** *p* < 0.01, *** *p* < 0.001, and **** *p* < 0.0001. Scale bar: 0.5 cm.

**Figure 8:**
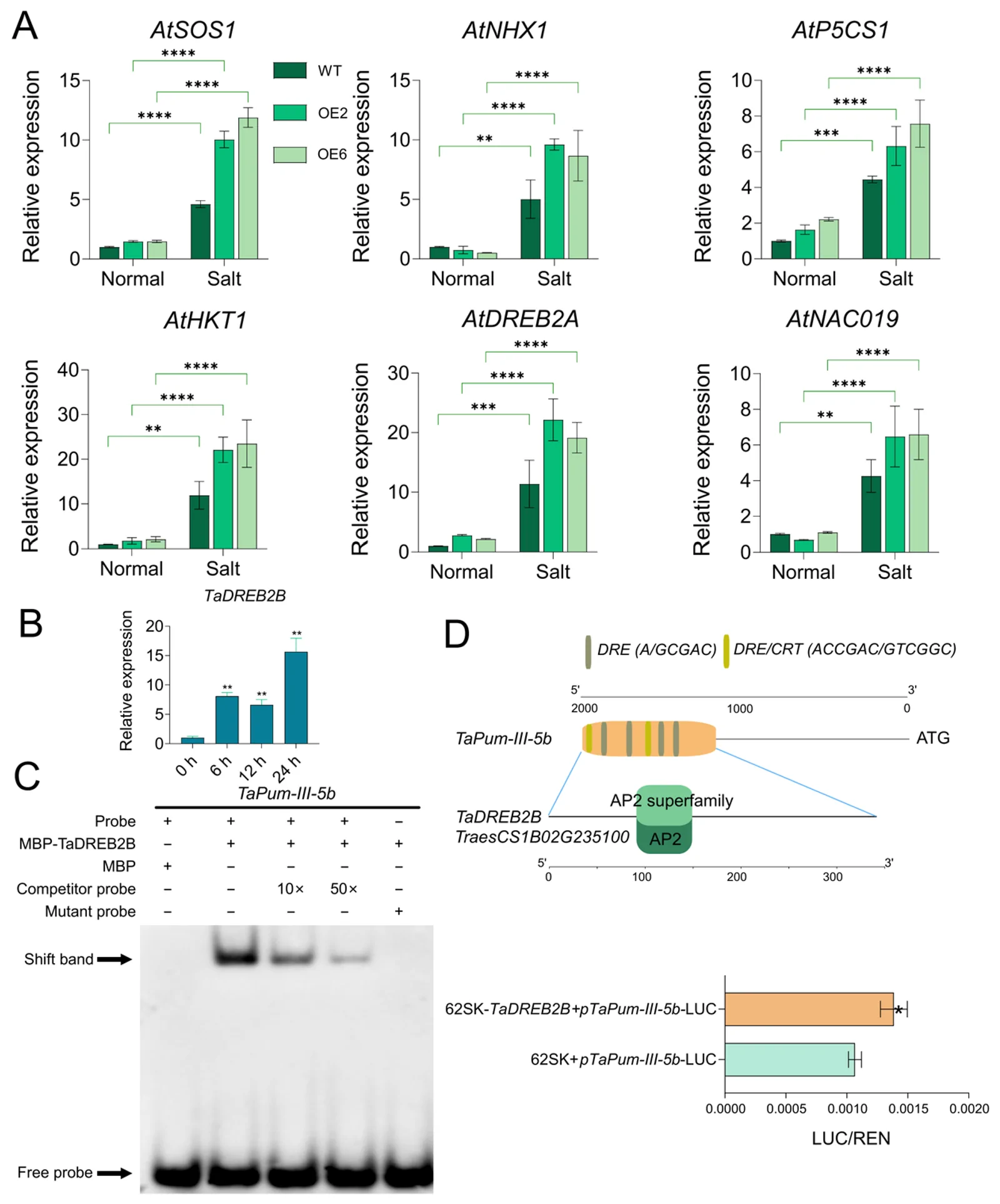
Molecular mechanisms underlying *TaPum-III-5b*-mediated salt tolerance. (**A**) Relative transcript levels of salt-responsive genes in WT and *TaPum-III-5b*-overexpressing lines (OE2 and OE6) under normal and salt-stress conditions, as determined by quantitative reverse transcription polymerase chain reaction (qRT-PCR). (**B**) Relative transcript levels of *TaDREB2B* in leaves of Luyan951 seedlings at 0, 6, 12, and 24 h after treatment with 200 mM NaCl, as determined by qRT-PCR. Gene-specific primers are listed in Supplementary Table S5. (**C**) Electrophoretic mobility shift assay (EMSA) of maltose-binding protein (MBP)-*TaDREB2B* binding to the *TaPum-III-5b* promoter dehydration-responsive element (DRE) probe. Lane 1, 5′-biotin-labeled WT probe with the MBP tag-only control; lane 2, WT probe with MBP-*TaDREB2B*; lanes 3 and 4, WT probe with MBP-*TaDREB2B* and 10- or 50-fold excess unlabeled WT competitor, respectively; lane 5, 5′-biotin-labeled mutant probe with MBP-*TaDREB2B*. (**D**) Schematic representation of the DRE cis-acting elements in the *TaPum-III-5b* promoter and Dual-LUC reporter assay of *TaDREB2B*-mediated promoter activation. *TaDREB2B* belongs to the AP2/ERF superfamily. The firefly luciferase/Renilla luciferase (LUC/REN) ratio represents the relative activity of the *TaPum-III-5b* promoter. Quantitative data are presented as the mean and standard deviation (SD) of at least three independent biological replicates. Asterisks indicate significant differences (* *p* < 0.05; ** *p* < 0.01; *** *p* < 0.001; **** *p* < 0.0001).

## Data Availability

The original contributions presented in this study are included in the article/Supplementary Material. Further inquiries can be directed to the corresponding author.

## Supplementary Materials

The following supporting information can be downloaded at: https://www.mdpi.com/article/10.3390/plants15192912/s1. Supplementary Data S1: MUSCLE alignment of the 101 Pum protein sequences used for phylogenetic analysis; Supplementary Data S2: Maximum-likelihood phylogenetic tree in Newick format. Figure S1: Predicted three-dimensional structures of representative Pum proteins from *Arabidopsis*, wheat, and maize; Figure S2: Chromosomal distribution of Pum genes in the wheat genome; Figure S3: Molecular identification and relative expression analysis of transgenic *Arabidopsis* lines overexpressing the wheat gene *TaPum*-III-5b; Figure S4: Melt-curve analysis of the *AtSOS1* and *AtNAC019* qRT-PCR products; Table S1: ID of the Pum family genes in wheat; Table S2: Analysis of physicochemical properties of Pum family members in wheat; Table S3: Duplication category and Ka/Ks analysis of *TaPum* gene pairs; Table S4: Predicted cis-acting elements in wheat Pum genes (*TaPum*); Table S5: Sequences of gene primers; Table S6: Representative Pum proteins and SWISS-MODEL structural-model quality metrics.
