# Supplementary material for: Genome-Wide Characterization of the Wheat Pum Gene Family and Heterologous Functional Analysis of TaPum-III-5b in Arabidopsis Under Salt Stress
Source: Plants (Basel). 2026 Sep 23;15(19):2912. doi: 10.3390/plants15192912 (PMC13666708; doi:10.3390/plants15192912)
Supplement: Supplementary file 1 [file plants-15-02912-s001.zip › Supplementary Files/Supplementary Figure 1 Predicted three-dimensional structures of representative Pum proteins from Arabidopsis, wheat, and maize.pdf]

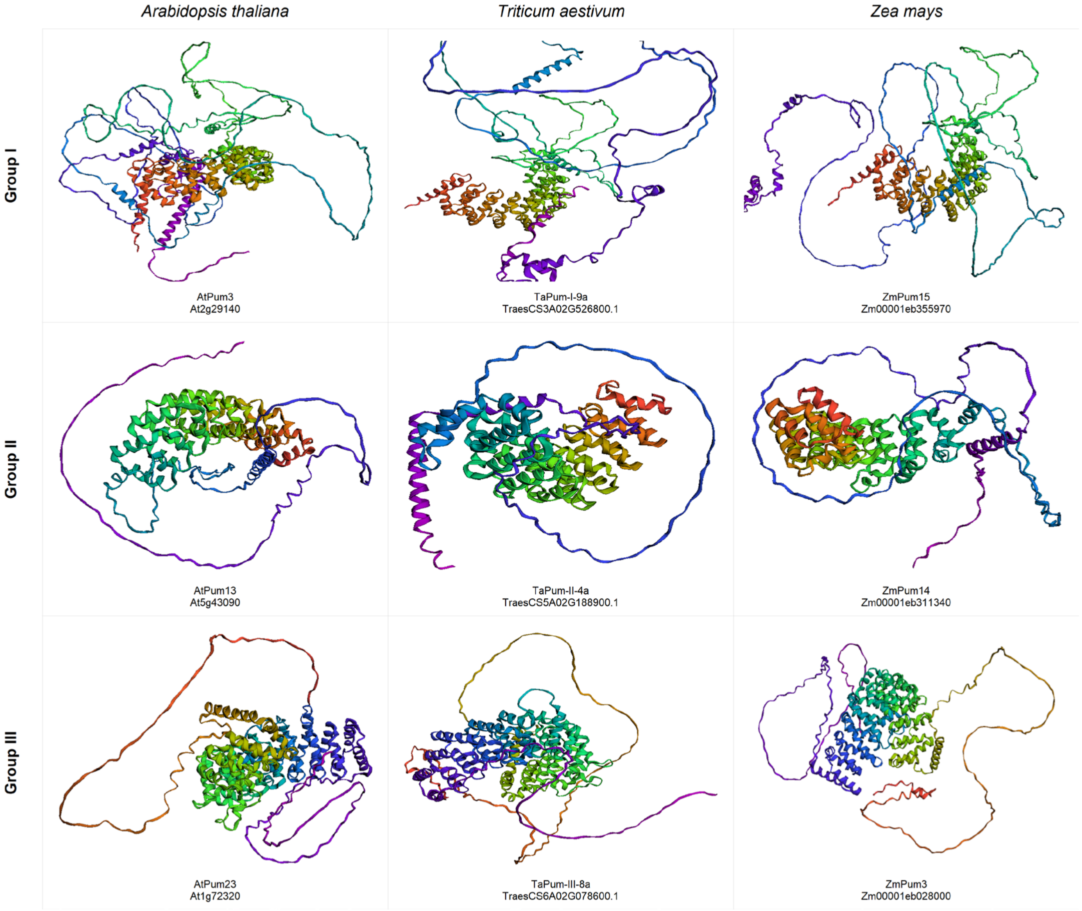

Figure S1. Predicted three-dimensional structures of representative Pum proteins from *Arabidopsis*, wheat, and maize. One representative protein from each phylogenetic group and species was selected for structural modelling with SWISS-MODEL. Rows correspond to Groups I-III, and columns correspond to *Arabidopsis thaliana*, *Triticum aestivum*, and *Zea mays*. Protein identifiers are shown below each model; templates, sequence identity, coverage, model-quality metrics, and Pum-HD positions are provided in Supplementary Table S6. Ribbon colors progress from the N terminus (blue) to the C terminus (red) and indicate residue position along the protein sequence, not secondary-structure type.
