# Supplementary material for: Genome-Wide Characterization of the Wheat Pum Gene Family and Heterologous Functional Analysis of TaPum-III-5b in Arabidopsis Under Salt Stress
Source: Plants (Basel). 2026 Sep 23;15(19):2912. doi: 10.3390/plants15192912 (PMC13666708; doi:10.3390/plants15192912)
Supplement: Supplementary file 1 [file plants-15-02912-s001.zip › Supplementary Files/Supplementary Figure 2 Chromosomal distribution of Pum genes in the wheat genome.pdf]

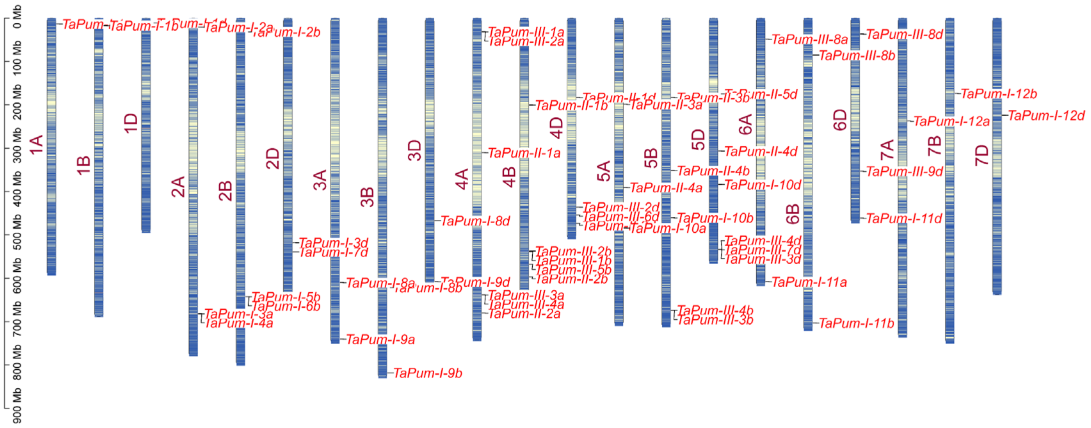

Figure S2. Chromosomal distribution of *Pum* genes in the wheat genome. The scale bar on the left indicates chromosome length in megabases (Mb). The color gradient on the chromosomes represents gene density, with darker blue indicating lower gene density.
