# Supplementary material for: Genome-Wide Characterization of the Wheat Pum Gene Family and Heterologous Functional Analysis of TaPum-III-5b in Arabidopsis Under Salt Stress
Source: Plants (Basel). 2026 Sep 23;15(19):2912. doi: 10.3390/plants15192912 (PMC13666708; doi:10.3390/plants15192912)
Supplement: Supplementary file 1 [file plants-15-02912-s001.zip › Supplementary Files/Supplementary Figure 3 Molecular identification and relative expression analysis of transgenic Arabidopsis lines overexpressing the wheat gene TaPum-III-5b.pdf]

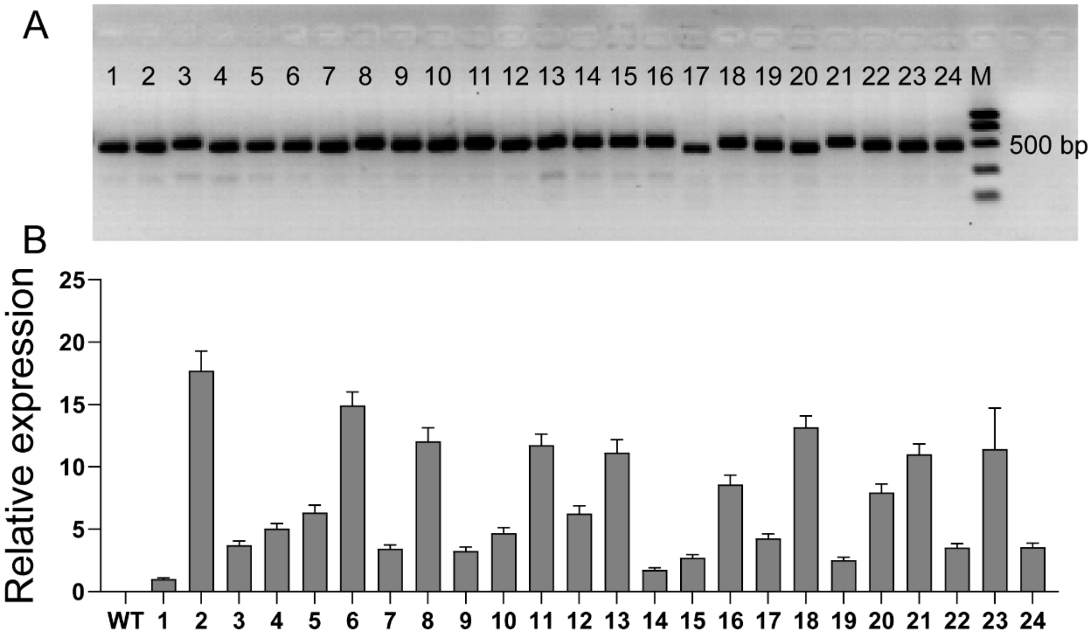

Figure S3. Molecular identification and relative expression analysis of transgenic *Arabidopsis* lines overexpressing the wheat gene *TaPum-III-5b*. (A) PCR verification of positive transgenic lines. (B) Relative expression levels of *TaPum-III-5b* in the transgenic lines. Numbers 1–24 denote the overexpression lines over1–over24, respectively. WT, wild type; M, DNA marker.
