# Supplementary material for: Genome-Wide Characterization of the Wheat Pum Gene Family and Heterologous Functional Analysis of TaPum-III-5b in Arabidopsis Under Salt Stress
Source: Plants (Basel). 2026 Sep 23;15(19):2912. doi: 10.3390/plants15192912 (PMC13666708; doi:10.3390/plants15192912)
Supplement: Supplementary file 1 [file plants-15-02912-s001.zip › Supplementary Files/Supplementary Figure 4 Melt-curve analysis of AtSOS1 and AtNAC019 qRT-PCR products.pdf]

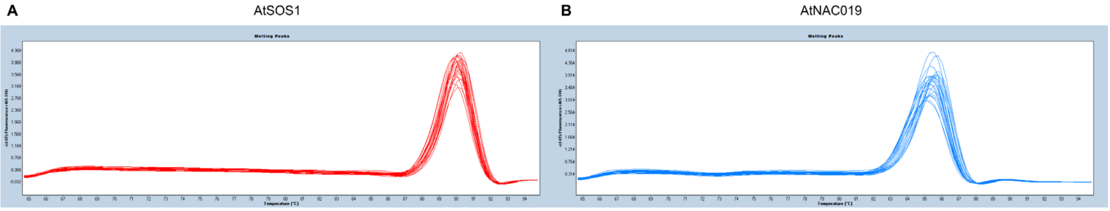

Figure S4. Melt-curve analysis of the qRT-PCR products. (A) Melt curve of the *AtSOS1* amplicon. (B) Melt curve of the *AtNAC019* amplicon. Each primer pair produced a single distinct melting peak, supporting the specificity of the qRT-PCR amplification.
